# Neural correlates of math anxiety – an overview and implications

**DOI:** 10.3389/fpsyg.2015.01333

**Published:** 2015-09-01

**Authors:** Christina Artemenko, Gabriella Daroczy, Hans-Christoph Nuerk

**Affiliations:** ^1^LEAD Graduate School, Eberhard Karls University of TuebingenTuebingen, Germany; ^2^Department of Psychology, Eberhard Karls University of TuebingenTuebingen, Germany; ^3^Leibniz Institute for Knowledge Media Research CenterTuebingen, Germany

**Keywords:** math anxiety, math performance, processing efficiency, emotion regulation, negative emotions

## Abstract

Math anxiety is a common phenomenon which can have a negative impact on numerical and arithmetic performance. However, so far little is known about the underlying neurocognitive mechanisms. This mini review provides an overview of studies investigating the neural correlates of math anxiety which provide several hints regarding its influence on math performance: while behavioral studies mostly observe an influence of math anxiety on difficult math tasks, neurophysiological studies show that processing efficiency is already affected in basic number processing. Overall, the neurocognitive literature suggests that (i) math anxiety elicits emotion- and pain-related activation during and before math activities, (ii) that the negative emotional response to math anxiety impairs processing efficiency, and (iii) that math deficits triggered by math anxiety may be compensated for by modulating the cognitive control or emotional regulation network. However, activation differs strongly between studies, depending on tasks, paradigms, and samples. We conclude that neural correlates can help to understand and explore the processes underlying math anxiety, but the data are not very consistent yet.

## Math anxiety

Math anxiety is important in psychological research due to its consequences: avoidance of future mathematics related career (Ashcraft, 2002) and course choices (Chipman et al., 1992) or situations containing mathematics even in daily life context (Kohn et al., 2013). In the PISA 2012 study, overall 59% of students reported worrying that it will be difficult for them in mathematics classes, and 30% feel helpless when doing a mathematics problem (OECD, 2013). According to a definition by Richardson and Suinn (1972), math anxiety “involves feelings of tension and anxiety that interfere with the manipulation of numbers and the solving of mathematical problems in a wide variety of ordinary life and academic situations”. It arises from unpleasant memories (Ma and Xu, 2004; Rubinsten and Tannock, 2010) and is related to math ability perception (Meece et al., 1990), self-regulation, and self-efficiency processes (Jain and Dowson, 2009) as well as to pedagogical factors (Rayner et al., 2009) and gender. For instance, girls generally report higher levels of math anxiety than boys (Devine et al., 2012).

Math anxiety is considered a multidimensional construct. One of the most well-known questionnaires – the mathematics anxiety rating scale (MARS) – differentiates between math test anxiety and numerical anxiety factors (Suinn and Winston, 2003). Besides this differentiation, the two dimensions most often confirmed are affective (emotional) and cognitive (worry) (Ho et al., 2000; Hopko, 2003; Harari et al., 2013). Furthermore, other factors such as behavioral, situational and physiological levels (Hembree, 1990; Krinzinger et al., 2009) may also play a role.

Math anxiety shares properties and mechanisms with test anxiety and general anxiety, but can also be distinguished from them (Baloglu, 1999; Kazelskis et al., 2000). Like other anxieties, high demands on cognitive resources and working memory capacities may moderate the relationship between anxiety and test performance (Owens et al., 2012). However, few studies control for other anxieties.

**FIGURE 1:**
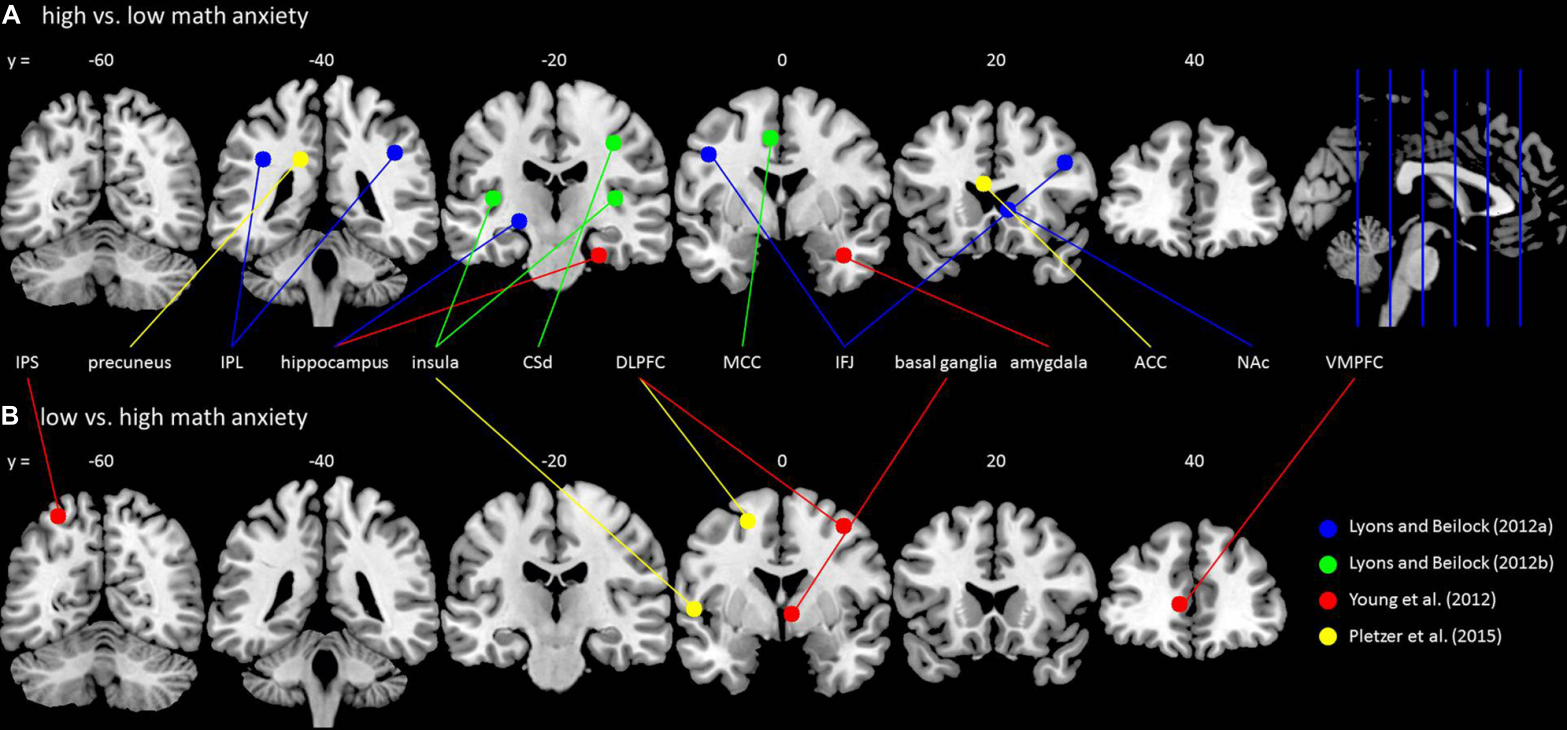
**Brain activation differences for math anxiety. (A)** Brain areas showing higher activation in high math-anxious individuals compared to low math-anxious individuals. **(B)** Brain areas showing higher activation in low math-anxious individuals compared to high math-anxious individuals^1^. The circles are centered around the activation maximum of each cluster with a radius of 5 mm and are located on the *y*-slice next to its *y*-value (e.g., the slide with a *y*-value of 0 contains all activation maxima from –10 ≤ *y* ≤ 10) by using the software MRIcron (www.mricro.com/mricron). Different studies are indicated by different colors: blue – Lyons and Beilock (a); green – Lyons and Beilock (2012b); red – Young et al. (2012); yellow – Pletzer et al. (2015). Abbreviations are adapted from the original studies: ACC, anterior cingulate gyrus; CSd, dorsal segment of central sulcus; DLPFC, dorsolateral prefrontal cortex; IFJ, inferior frontal junction; IPL, inferior parietal lobe; IPS, intraparietal sulcus; MCC, midcingulate cortex; NAc, nucleus accumbens; VMPFC, ventromedial prefrontal cortex.

In summary, math anxiety is a common phenomenon which has considerable impact on the performance in math tasks (e.g., Ashcraft and Kirk, 2001). A solid neuroscientific understanding would provide better perspectives for interventions and therapies, but the underlying neurocognitive mechanisms are still unclear. In this mini review, we provide an overview of recent literature addressing the issue of math anxiety from a neuroscientific perspective (cf. **Figure 1, Table 1**). We will first address the neural correlates of the affective component of math anxiety and its regulation. Then, we turn to the neural correlates of the cognitive components of math anxiety, in particular processing efficiency. Finally, we outline how math anxiety and its neural correlates are related to math performance and finish with future perspectives.

**Table 1:** Selected papers focusing on the neural correlates of math anxiety.

Study	Sample	Math anxiety	Groups	Tasks	Controlled variables	Results
**fMRI studies**						
Lyons and Beilock (2012a)	28 adults	sMARS	20% (out of *N* = 108)	Single-digit arithmetic verification task (simple/ complex, subtraction/ multiplication); control word task; cues before task	Trait anxiety, working memory	High math-anxious individuals show with decreasing math deficits increasing differences in IFJ and IPL before the math task and in right NAc and left hippocampus during math task (increasing emotion regulation leads to compensation of math deficit)
Lyons and Beilock (2012b)	28 adults	sMARS	ca. 50 %	Single-digit arithmetic verification task (simple/ complex, subtraction/ multiplication); control word task; cues before task	Trait anxiety, working memory	High math-anxious individuals show higher activation in bilateral dorso-posterior insula, MCC and right CSd (pain-related activity before math task)
Young et al. (2012)	46 7- to 9-year-old children	SEMA	50%	Single-digit arithmetic verification task (simple/ complex; addition/ subtraction); control number identification and passive fixation task	IQ, working memory, reading ability, math ability, trait anxiety	High math-anxious individuals show hyperactivity and abnormal effective connectivity of right amygdala extending into anterior hippocampus (processing negative emotions), less activation in IPS, right DLPFC, basal ganglia (less efficient task processing) and greater deactivation in VMPFC (emotion regulation)
Pletzer et al. (2015)	36 adults	MARS30-brief	25% (out of *N* = 127)	Two-digit number magnitude comparison task, number bisection task; control mental rotation task, control verbal reasoning task	Math ability	Low math-anxious individuals show moderately stronger deactivation within the task-related default mode network than high math-anxious individuals (less efficient processing), high math-anxious individuals did not activate left DLPFC, left inferior frontal gyrus and insula (task-irrelevant instead of task-relevant inhibitory control)
**tDCS study**						
Sarkar et al. (2014)	45 adults	Brief version of MARS	25% (out of *N* = 165)	Single-digit arithmetic verification task with affective primes (addition, subtraction, multiplication, division); control flanker task	Age, gender	Stimulating the DLPFC leads to better math performance and decreased cortisol concentrations in high math-anxious individuals (less stress), but impaired math performance and prevented cortisol decrease in low math-anxious individuals
**ERP studies**						
Suárez-Pellicioni et al. (2013a)	26 adults	sMARS	25% (out of *N* = 342)	Single-digit arithmetic verification task (split effect; addition)	Math ability, trait anxiety, spatial visualization, reasoning ability, verbal comprehension ability, gender distribution	High math-anxious individuals show an enhanced and delayed P600/3b for large-split solutions (difficulty in inhibiting processing of irrelevant information and less processing efficiency)
Suárez-Pellicioni et al. (2013b)	34 adults	sMARS	25% (out of *N* = 452)	Single-digit numerical Stroop task; control classical Stroop task	Trait and state anxiety, years of formal education, age, handedness, ethnicity, gender distribution	High math-anxious individuals show an enhanced ERN (abnormal error monitoring), but no difference for CRN or Pe (normal generic response monitoring processes)
Suárez-Pellicioni et al. (2014)	34 adults	sMARS	25% (out of *N* = 490)	Single-digit numerical Stroop task	Trait and state anxiety, simple math ability, years of formal education, age, handedness, ethnicity, gender distribution	High math-anxious individuals show a tendency for an enhanced conflict sustained potential (stimulus conflict processing), no enhanced N450 and greater conflict sustained potential amplitude in conflict adaptation (attentional control deficit and distractibility)
Núñez-Peña and Suárez-Pellicioni (2014)	53 adults	sMARS	25% (out of *N* = 629)	Single-digit number magnitude comparison task (size effect, distance effect)	Trait anxiety, age, years of formal education, gender distribution, handedness, ethnicity	High math-anxious individuals show an enhanced ERP distance and size effect (less precise number magnitude representation)

*The studies are sorted by method and include information on the analyzed sample size, the math anxiety questionnaire used, the cut-off value for a possible prescreening group for low vs. high math-anxious individuals, the numeric and the control task, controlled or matched variables between groups and the main results of the study.*

*ACC, anterior cingulate gyrus; CRN, correct-response negativity; CSd, dorsal segment of central sulcus; DLPFC, dorsolateral prefrontal cortex; ERN, error-related negativity; ERP, event-related potential; fMRI, functional magnetic resonance imaging; IFJ, inferior frontal junction; IPL, inferior parietal lobe; IPS, intraparietal sulcus; IQ, intelligence quotient; MCC, midcingulate cortex; MARS, math anxiety rating scale; NAc, nucleus accumbens; Pe, error-related positivity; SEMA, scale for early mathematics anxiety; tDCS, transcranial direct current stimulation; VMPFC, ventromedial prefrontal cortex.*

## Affective Response in Math Anxiety

The affective component of math anxiety addresses the actual feelings and physiological reactions elicited by a math task in high math-anxious individuals. Thus, individuals with math anxiety reported negative attitudes such as dislike toward mathematics (Cornell, 1999), negative emotions such as tension (Richardson and Suinn, 1972), frustration (Hembree, 1990), and emotions related to learning outcomes such as shame, hopelessness (Pekrun et al., 2002). On a neural level, two networks representing the emotionality of math anxiety could be found: the pain network involving the insula (Lyons and Beilock, 2012b) and the fear network centered around the amygdala (Young et al., 2012).

Regarding the first, math anxiety elicited increased activation in the pain perception network including the bilateral dorso-posterior insula and mid-cingulate cortex (Lyons and Beilock, 2012b). The insula is supposedly associated with the subjective feeling of visceral threat for the upcoming math task and relief when confronted with a non-math task. The mid-cingulate cortex was not selective for pain perception *per se* but reflected similar emotionality. The pain-related activity was observed when high math-anxious individuals faced a math task but not during the math task itself, explaining that high math-anxious individuals try to avoid math. Additional analyses confirmed that math anxiety and not differences in math performance was responsible for the affective component.

Regarding the fear network, high math-anxious children showed hyperactivity and abnormal effective connectivity in the right basolateral amygdala (Young et al., 2012). Since the amygdala is known for fear perception, its activation during a math task confirms the children’s fear of math. Moreover, the aberrant connectivity of the amygdala is reflected by a greater connectivity to the ventromedial prefrontal cortex in order to facilitate compensatory mechanisms for performance and by a reduced connectivity to the bilateral superior parietal lobule, leading to the performance deficit.

In summary, the affective component of math anxiety is associated with pain-related activity before math tasks and fear-related activity during math tasks, independent of trait anxiety. However, are both networks active in the same math-anxious individual or is this age-dependent? Since evidence for pain-related activity was found in adults and evidence for fear-related activity was found in children, the data point to an age-dependency of recruited networks which should be systematically studied with the same paradigms.

## Emotion Regulation in Math Anxiety

Since math anxiety elicits negative emotional responses to math, high math-anxious individuals need to process and regulate these emotions which lead to cognitive consequences (Rubinsten and Tannock, 2010). Consequently, working memory is occupied with the math-related anxiety and less resources are available for the math task, resulting in impaired math performance (Ashcraft and Kirk, 2001, Ashcraft and Krause, 2007). The detrimental effect of math anxiety on performance is mediated by working memory and emotion regulation (Hopko et al., 1998; Ashcraft and Krause, 2007).

On the neural level, brain connectivity and brain activity patterns are altered by math anxiety due to emotion regulation. For instance, high math-anxious children showed a greater coupling of the hyperactive amygdala with cortical regions involved in processing and regulating negative emotions during the math task (Young et al., 2012). This led to greater deactivation in the ventromedial prefrontal cortex compared to their low math-anxious counterparts. Furthermore, the typical neural activation within the left inferior frontal gyrus and the insula for the processes of place-value integration in multi-digit numbers was absent in high math-anxious individuals (Pletzer et al., 2015). These areas are associated with inhibitory control during the number comparison task. The results, therefore, suggest that math anxiety inhibits emotional processing within task-irrelevant areas instead of activating task-relevant inhibitory control regions (Pletzer et al., 2015). Thus, math-anxious individuals seemingly focus on their math-related emotions during the task rather than on the task itself which can be detrimental for their task performance. However, the neuroscientific literature does not hint at the strategies involved in emotion regulation (cf. Gross and John, 2003).

Controlling math-related emotions in math-anxious individuals does not automatically result in performance impairment. Emotion regulation can even help prevent or at least minimize the impact of an anxiety-caused performance deficit. Lyons and Beilock (2012a) observed that high math-anxious individuals who used the fronto-parietal network associated with cognitive control and emotion regulation before the math task, compensated for their math-related deficit. This network, consisting of the bilateral inferior frontal junction and the bilateral inferior parietal lobe, is associated with high-level cognitive control processes. When high math-anxious individuals ramp up these resources before the math task starts, activation in the right nucleus accumbens and the left hippocampus, associated with motivating behavior, and integration of cognitive control, is increased during math performance. (Lyons and Beilock, 2012a; Young et al., 2012). Consequently, they show almost no math deficit despite their math anxiety.

The dorsolateral prefrontal cortex (DLPFC), as one part of this fronto-parietal network (Lyons and Beilock, 2012a), seems critical for the math anxiety-induced mediation of emotion regulation on math performance. When performance in the math task was not controlled for, high math-anxious individuals showed reduced activity in the right DLPFC and the bilateral basal ganglia associated with working memory and attention (Young et al., 2012). When performance was controlled for, the response to the compatibility effect was reduced in the left DLPFC (Pletzer et al., 2015). When processing in the DLPFC was enhanced by applying transcranial direct current stimulation (tDCS), high math-anxious individuals showed improved performance in a simple arithmetic task and less stress during the math task as indicated by decreased cortisol concentrations (Sarkar et al., 2014). Interestingly, the same stimulation protocol had the opposite effect on individuals with low math anxiety – arithmetic performance was impaired and cortisol decrease was prevented. Taken together, math anxiety is associated with reduced DLPFC activity independent of math performance, but by facilitating processing within the DLPFC, the negative emotional reaction to math can be reduced and thus math performance improves.

To sum up, math anxiety elicits a negative emotional response to the math task, usually leading to impaired performance because of the additional involvement in emotion regulation. However, by enhancing the capacity for emotion regulation or cognitive control, high math-anxious individuals can compensate for their math-specific performance deficit. This influence of math anxiety on performance in arithmetic tasks can be conceptualized in a more general theoretical framework addressing the impact of anxiety on processing efficiency.

## Impact of Math Anxiety on Processing Efficiency

Anxiety is hypothesized to have a general influence on processing efficiency (cf. processing efficiency theory, Eysenck and Calvo, 1992; and its extension: attentional control theory, Eysenck et al., 2007). Performance efficiency is the relationship between performance effectiveness (the quality of performance) and processing efficiency (the use of processing resources). According to both theories, “anxiety impairs processing efficiency more than performance effectiveness” though “impairing the efficiency of the central executive component of the working memory system” (Derakshan and Eysenck, 2009). In the attentional control theory (where attentional control refers to an individual’s capacity to choose what to pay attention to), it is assumed that anxiety impairs both positive and negative attentional control. Attentional and processing resources are diminished by worry, and compensated by increased cognitive efforts. ERP studies (Suárez-Pellicioni et al., 2013a,b, 2014) and a neuro-imaging study (Pletzer et al., 2015) suggest that the processing efficiency hypothesis mentioned above can be applied to math anxiety and its relation to arithmetic performance.

First, it was shown that math anxiety influenced simple arithmetic within a verification task, although math ability and general anxiety were controlled for (Suárez-Pellicioni et al., 2013a). When the solution of the single-digit addition problem was dramatically incorrect and had to be rejected, the evoked P600/3b component was enhanced and delayed with increasing math anxiety. Thus, high math-anxious individuals have problems with inhibiting distractor-related processing and, therefore, need more resources and time to evaluate such solutions, i.e., math anxiety decreases processing efficiency in simple arithmetic tasks which is in line with the processing efficiency theory.

Second, math anxiety led to abnormal conflict monitoring and adaptation within a numerical Stroop task (Suárez-Pellicioni et al., 2013b, 2014). For instance, during the evaluation of errors, the error-related negativity (response-locked potential at 50–150 ms after the occurrence of an error) was enhanced, with no difference in behavioral performance (Suárez-Pellicioni et al., 2013b). This suggests that high math-anxious individuals have to increase their cognitive effort to compensate for fewer resources. Furthermore, math anxiety causes abnormal conflict adaptation: the early N450 potential is missing during conflict processing and subsequently the sustained conflict potential is increased, suggesting a rise of cognitive control to solve the conflict (Suárez-Pellicioni et al., 2014). Therefore, math anxiety is associated with a reactive recruitment of attentional control and increased distractibility to task-irrelevant information. This supports the attentional control theory, since anxiety is considered to reduce attentional resources to the task and thus cognitive effort has to be increased in order to reach comparable performance. Moreover, independent of general anxiety, it shows the specific effect of math anxiety on processing efficiency.

Finally, math anxiety reduced the deactivation of the default mode network which usually shows less activation during cognitive tasks (Pletzer et al., 2015). In particular, a moderately stronger deactivation within the task-related default mode network including the precuneus and the anterior cingulate gyrus was found in low compared to high math-anxious individuals. Since deactivation of the default mode network is an indicator of processing efficiency, math anxiety reduces processing efficiency in the math task and increases the effort to control the negative emotional response in order to achieve similar performance.

The neuroscientific findings support the idea that the processing efficiency theory can be applied to math anxiety. High math-anxious individuals show less efficient neural processing in numerical tasks and thus require more effort than low-anxious individuals to reach similar performance levels.

## Math Anxiety and Math Performance Deficit

Math anxiety considerably impacts performance in math tasks (Ma, 1999; Ashcraft and Kirk, 2001; Cates and Rhymer, 2003; Ashcraft and Moore, 2009). Several behavioral studies suggest that math anxiety especially impairs performance in difficult math tasks, as indicated by the anxiety–complexity effect (Ashcraft and Faust, 1994; Faust et al., 1996; Ashcraft and Kirk, 2001; Suárez-Pellicioni et al., 2013a). This implies that the more complex the arithmetic problem, the larger the impairment of the high math-anxious individuals. For instance, the detrimental effect of math anxiety on performance in an arithmetic task is larger for two-digit than for single-digit addition or for addition requiring a carry operation compared to addition not requiring a carry operation (Faust et al., 1996). However, more recent behavioral (Maloney et al., 2010, 2011) and ERP studies (Suárez-Pellicioni et al., 2013a; Núñez-Peña and Suárez-Pellicioni, 2014) provide evidence that math anxiety already affects basic number processing: number magnitude processing, place-value processing and simple arithmetic processing.

Essentially, high math-anxious individuals have a less precise number magnitude representation than their low math-anxious counterparts (Núñez-Peña and Suárez-Pellicioni, 2014). This was found in a symbolic number comparison task for both the distance and the size effect, reflected by increased amplitude of the positive peak in the difference wave around 200–250 ms and corroborated by increased reaction time differences on the behavioral level. This shows that already the underlying mechanisms of basic numerical processing are altered by math anxiety.

Math anxiety furthermore influences place-value processing within a number comparison task. Thus, the compatibility effect was accompanied by higher neural activation in the inferior frontal cortex in incompatible trials for low math-anxious individuals but not for high math-anxious individuals (Pletzer et al., 2015). The finding suggests a math anxiety-related failure when inhibitory functions related to the numerical stimuli are required and thus, basic place-value integration is not effective. This goes beyond behavioral studies on complex place-value integration such as the carry effect (Faust et al., 1996).

The neurocognitive effect of math anxiety on performance in simple arithmetic has already been shown in children (Young et al., 2012). Compared to their low math-anxious counterparts, high math-anxious children show less activation in the left intraparietal sulcus, superior parietal lobe and right DLPFC, i.e., in the fronto-parietal network responsible for numerical processing. This underactivation within the number processing network causes their math anxiety-related deficit in performance, reflected by marginally lower accuracy and less differentiation between RTs across difficulty levels. While the authors assume the neural effect to be independent of performance, this activation pattern could not be replicated in other studies (Lyons and Beilock, 2012a,b; Pletzer et al., 2015) and thus further research has to disentangle the confound of math anxiety and performance.

In conclusion, math anxiety affects the neural signatures of basic numerical effects, even when performance in the respective tasks is comparable. This shows that math anxiety not only hinders mathematical learning, causing a math deficit which can be observed in more complex math tasks, but also that the emotional response to math already alters basic number processing on a neural level. However, further research is needed to neurocognitively evaluate the impact of math anxiety on task difficulty.

## Conclusion and Perspectives

Neurocognitive studies suggest that math anxiety elicits an affective response within the fear and pain network in the brain. In order to deal with these negative emotions, brain areas associated with emotion regulation are active during math performance which may lead to limited capacities, impaired performance, and less efficient processing even in simple tasks. However, by extending these cognitive and emotional control capacities within the fronto-parietal brain network, high math-anxious individuals may still be able to compensate for the anxiety-related performance deficit. The neuroscientific literature suggests interventions which focus on controlling the negative emotional response to math (Lyons and Beilock, 2012a) to overcome the vicious circle of math anxiety and poor math performance.

The most important problem for research on math anxiety is that the neurocognitive activation patterns for math anxiety are confounded with math performance, since high math-anxious individuals usually perform worse in math tasks than their low anxious counterparts. When performance is not controlled for, the resulting effects of math anxiety could be due to this performance difference rather than due to math anxiety. Future research should, therefore, disentangle this confound by differentiating the math anxiety groups matched for math ability (cf. Pletzer et al., 2015) or using the interindividual variability in performance within the high math-anxious group (cf. Lyons and Beilock, 2012a). Note that the simple use of covariates may not be appropriate when relations between math anxiety and other variables are not linear.

Investigating the neurocognitive foundations of math anxiety can help explain the mechanisms that lead to performance deficits, detect anxiety-related differences in brain function, also in the absence of behavioral differences, and identify physiological markers of the emotional response to math. However, the few studies focusing on the neural correlates of math anxiety vary greatly in their methods (neuro-imaging, neurophysiological, and non-invasive brain stimulation), tasks (from simple numerical to complex arithmetic), and samples (adults, children). The methods differ in the investigation of correlational and causal structure–function relationships, the complexity of numerical tasks determines the degree of involvement of working memory resources and math ability levels depend on development. This leads to highly inconsistent results with little overlap between studies (cf. **Figure 1**). So far, this can be explained by differences in assessment, paradigms, and samples. Future research may address these issues by systematically manipulating methods, tasks, and samples to ensure that different results in different studies are due to methodological differences. Research on developmental trajectories could especially help identify the age-related neurocognitive correlates of math anxiety.

## Conflict of Interest Statement

The authors declare that the research was conducted in the absence of any commercial or financial relationships that could be construed as a potential conflict of interest.

## Footnotes

1 Note that further activation differences between high and low math-anxious individuals were observed in other brain areas such as the left anterior inferior frontal gyrus (Lyons and Beilock, 2012a), the supplementary motor area (Pletzer et al., 2015), the right primary somatosensory cortex (Young et al., 2012), and the dorsal segment of the right central sulcus (Lyons and Beilock, 2012b). But since the authors of the respective papers did not relate these activation differences to math anxiety, we did not include them in **Figure 1, Table 1** and the current review.
